# A Novel Source of Methylglyoxal and Glyoxal in Retina: Implications for Age-Related Macular Degeneration

**DOI:** 10.1371/journal.pone.0041309

**Published:** 2012-07-19

**Authors:** Kee Dong Yoon, Kazunori Yamamoto, Keiko Ueda, Jilin Zhou, Janet R. Sparrow

**Affiliations:** 1 Department of Ophthalmology, Columbia University, New York, New York, United States of America; 2 Department of Pathology and Cell Biology,Columbia University, New York, New York, United States of America; University of Florida, United States of America

## Abstract

Aging of retinal pigment epithelial (RPE) cells of the eye is marked by accumulations of bisretinoid fluorophores; two of the compounds within this lipofuscin mixture are A2E and all-*trans*-retinal dimer. These pigments are implicated in pathological mechanisms involved in some vision-threatening disorders including age-related macular degeneration (AMD). Studies have shown that bisretinoids are photosensitive compounds that undergo photooxidation and photodegradation when irradiated with short wavelength visible light. Utilizing ultra performance liquid chromatography (UPLC) with electrospray ionization mass spectrometry (ESI-MS) we demonstrate that photodegradation of A2E and all-*trans*-retinal dimer generates the dicarbonyls glyoxal (GO) and methylglyoxal (MG), that are known to modify proteins by advanced glycation endproduct (AGE) formation. By extracellular trapping with aminoguanidine, we established that these oxo-aldehydes are released from irradiated A2E-containing RPE cells. Enzyme-linked immunosorbant assays (ELISA) revealed that the substrate underlying A2E-containing RPE was AGE-modified after irradiation. This AGE deposition was suppressed by prior treatment of the cells with aminoguanidine. AGE-modification causes structural and functional impairment of proteins. In chronic diseases such as diabetes and atherosclerosis, MG and GO modify proteins by non-enzymatic glycation and oxidation reactions. AGE-modified proteins are also components of drusen, the sub-RPE deposits that confer increased risk of AMD onset. These results indicate that photodegraded RPE bisretinoid is likely to be a previously unknown source of MG and GO in the eye.

## Introduction

Several histopathological changes in the retinal pigment epithelial cell (RPE) and in its underlying basement membrane (Bruch’s membrane), are distinctly characteristic of aging and may contribute to sight-threatening age-related macular degeneration (AMD). For instance, aging of RPE is associated with a progressive accumulation of autofluorescent pigments (lipofuscin) consisting of photo-sensitive bisretinoid compounds [1]. In Bruch’s membrane, there is a build-up of esterified cholesterol-rich apolipoprotein B-containing lipoprotein that originates from RPE cells [2]. Bruch’s membrane also undergoes thickening, diffusional rates across this layer are diminished [3], the integrity of the elastic lamina of Bruch’s membrane is compromised [4] and collagens in this layer become cross-linked and less soluble [5]. Histologically visible dome-shaped extracellular deposits (drusen) that can be detected as yellow-white lesions in a retinal fundus image, are also common in older individuals. Drusen size and area within the macula are factors considered in the clinical characterization of age-related macular degeneration [6]. Besides containing neutral lipid, drusen house a number of proteins which function within the complement system [7]. This feature is of interest since genetic studies demonstrate that sequence variants in some complement related proteins confer increased risk or protection against age-related macular degeneration (AMD) [8]–[13].

**Figure 1:**
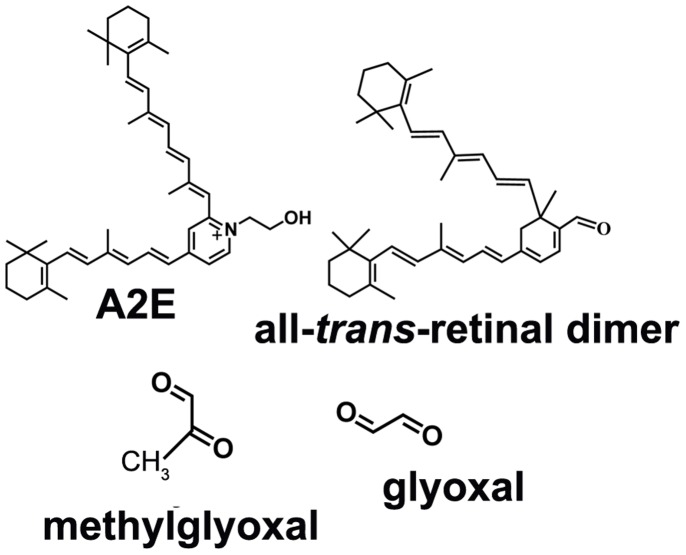
Structures of the bisretinoids A2E and all-*trans*-retinal dimer; and the oxo-aldehydes, methylglyoxal and glyoxal.

As part of the pathological process, resident proteins within drusen accumulate non-enzymatic modifications in the form of advanced glycation end-products (AGEs). AGE-modified proteins have been detected in drusen by immunocytochemistry, by Raman confocal microscopy and by chromatography [14]–[18]. AGE formation is pronounced in diabetes and several disorders of aging such as atherosclerosis. In diabetes, AGE modification is a product of autooxidation and decomposition of carbohydrates and is considered to be a major pathogenic link between hyperglycemia and the onset and progression of disease [19]. Conversely, the origin of AGEs such as carboxymethyllysine (CML) and carboxyethyllysine (CEL) in ocular drusen is not known. Here we have demonstrated that methylglyoxal (MG) and glyoxal (GO) (Figure 1), two agents known to form AGEs, are released upon photodegradation of A2E and all-*trans*-retinal dimer, two bisretinoids that accumulate as lipofuscin in RPE. Bisretinoid cleavage, upon exposure to wavelengths of light that reach the retina, represents a previously unrecognized source of these dicarbonyls. While various processes play a role in Bruch’s membrane changes and drusen formation, these findings are indicative of a contribution from lipofuscin photooxidation and cleavage in RPE.

## Results

### Detection of MG and GO as Photodegradation Products of A2E and all-*trans*-retinal Dimer. Reaction with 4NPH

**Figure 2:**
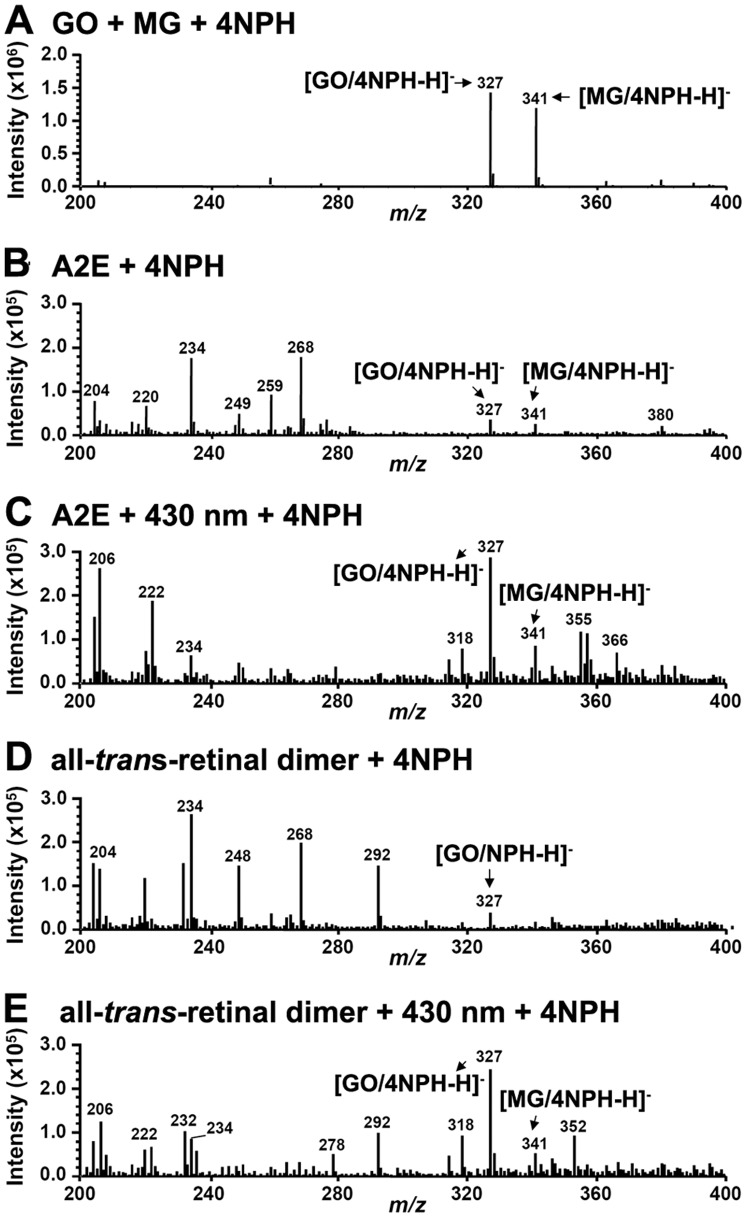
Irradiation (430 nm) of A2E and all*-trans*-retinal dimer leads to production of glyoxal (GO) and methylglyoxal (MG). Negative ESI-MS spectra in the range *m/z* 200–400. Detection of GO and MG by reaction with 4NPH. (*A*) Authentic standards. Incubation of 4NPH with GO and MG (obtained commercially) yielded products at *m/z* 327 ([GO/4NPH-H]^-^) and *m/z* 341 ([MG/4NPH-H]^-^), respectively. (*B*) Incubation of 4NPH with A2E. (*C*) Incubation of 4NPH with irradiated A2E. (*D*) Incubation of 4NPH with all-t*rans*-retinal dimer. (*E*) Incubation of 4NPH with irradiated all*-trans*-retinal dimer. Augmentation of the *m/z* 327 and *m/z* 341 peaks in the irradiated samples is indicative of photodegradation-associated release of GO and MG.

To acquire evidence for the liberation of GO and MG when the bisretinoids A2E and all-*trans*-retinal dimer undergo photooxidation-elicited photodegradation, we trapped these volatile dicarbonyl fragments with 4NPH, a compound known to readily react with carbonyl moieties. As demonstrated in Figure 2, reaction of 4NPH with commercially available MG (*m/z* 72) and GO (*m/z* 58) generated the expected products at *m/z* 327 ([GO/4NPH-H]^−^) ([M-H]^−^) and *m/z* 341 ([MG/4NPH-H]^−^) ([M-H]^−^) [2-(4-nitrophenyl)hydrazone], respectively. Subsequently, samples of A2E and all-*trans*-retinal dimer were irradiated (430 nm) under conditions known to result in their photooxidation and photodegradation [20]–[22]. The samples were then incubated with 4NPH and analyzed by negative mode ESI-MS. The reaction yielded peaks at *m/z* 327 and *m/z* 341 (Figure 2 C, E) that were considerably magnified relative to non-irradiated samples (Figure 2, B and D) and that were indicative of the presence of GO and MG, respectively, in the photodegradative mixtures. These adducts were generated when assaying at both 60°C and room temperature. The generation of these peaks could be explained by the facile reaction of 4NPH with the photo-products GO and MG, that were released after photooxidation and photodegradation of the bisretinoids. Since we employed a cell-free assay, the MG and GO detected was not attributable to the degradation of other organic compounds such as glyceraldehyde-3-phosphate [23].

### MG and GO Released upon Bisretinoid Photodegradation Form Adducts with Aminoguanidine

**Figure 3:**
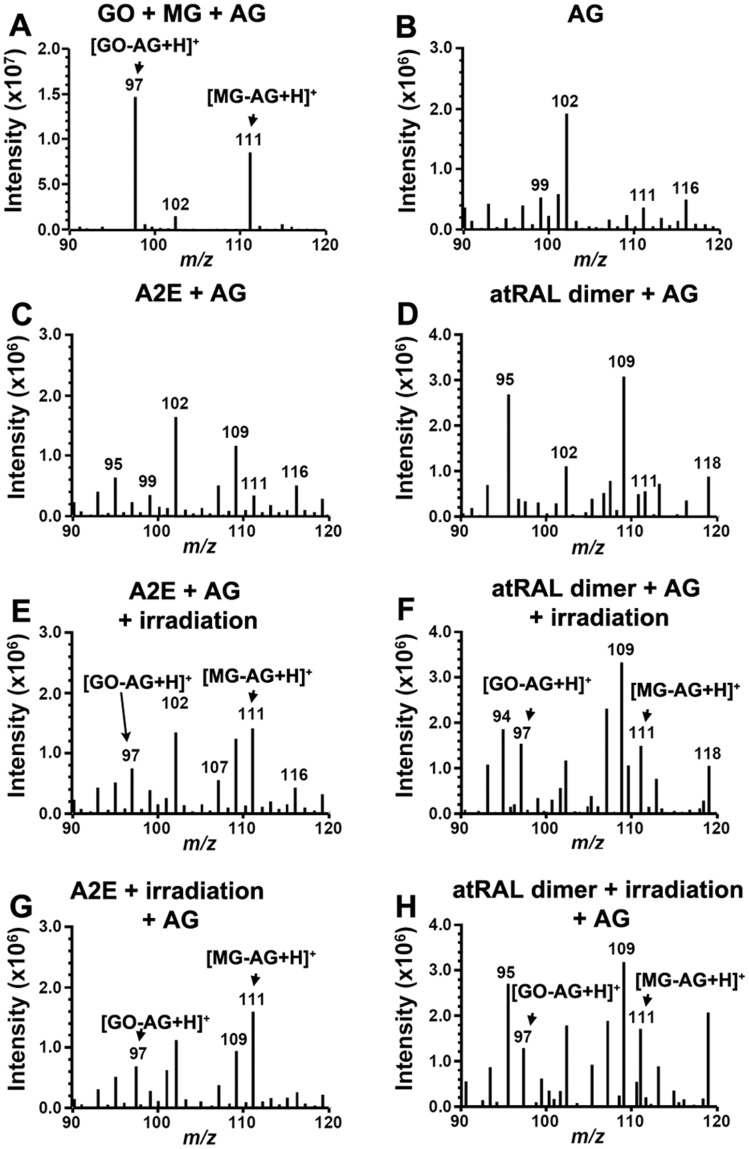
Methylglyoxal (MG) and glyoxal (GO) released by photodegradation of A2E and all-*trans*-retinal dimer (atRAL dimer) forms adducts with aminoguanidine (AG). Positive ESI-MS spectra in the range *m/z* 90–120 to detect GO-AG adduct (*m/z* 97; [M+H]^+^) and MG-AG adduct (*m/z* 111; [M+H]^+^). (*A*) AG reaction with commercial glyoxal (GO) and methylglyoxal (MG). (*B*) AG alone. Note the absence of *m/z* 97 and *m/z* 111. (*C*) A2E and AG (no irradiation). (*D*) atRAL dimer and AG (no irradiation). (*E*) Irradiation of a mixture of A2E and AG. (*F*) Irradiation of a mixture of atRAL dimer and AG. (*G*) Incubation of photooxidized A2E (A2E + irradiation) and AG. (*H*) Incubation of photooxidized all-*trans*-retinal dimer (atRAL dimer + irradiation) and AG.

Another compound with MG and GO scavenging capability is aminoguanidine (*m/z* 74), a small molecule that was initially designed to therapeutically inhibit AGE-modification of proteins [24]. Thus to further test for release of MG (*m/z* 72) and GO (*m/z* 58) upon photodegradation of A2E and all-*trans*-retinal dimer, we also incubated aminoguanidine with irradiated A2E and all-*trans*-retinal dimer. Reaction of aminoguanidine with commercially obtained GO and MG demonstrated production of the expected adducts at *m/z* 97 ([M+H]^+^; GO-derivatized aminoguanidine) and *m/z* 111 ([M+H]^+^; MG-derivatized aminoguanidine, 3-amino-5/6-methyl-1,2,4-triazine) (Figure 3A) [24]. The GO-AG adduct that we detected had the same molecular weight as authentic 3-amino-1,2,4-triazine (Figure S1). These *m/z* species were negligible in samples of aminoguanidine alone (Figure 3B) and when non-irradiated A2E (Figure 3C) or non-irradiated all-*trans*-retinal dimer (Figure 3D) were analyzed. However, incubation of aminoguanidine with A2E (Figure 3, E and G) and all-*trans*-retinal dimer (Figure 3, F and H) during irradiation (Figure 3, E and F) or after irradiation (Figure 3, G and H) resulted in marked *m/z* 97 and *m/z* 111 ([M+H]^+^; MG-derivatized aminoguanidine, 3-amino-5/6-methyl-1,2,4-triazine) signals. These results were indicative of the release of GO and MG upon photooxidation-associated degradation of the bisretinoids. Again, since we utilized a cell-free assay, the MG and GO detected was not attributable to the degradation of other organic compounds [23].

### AGE Formation on Extracellular Fibronectin

**Figure 4:**
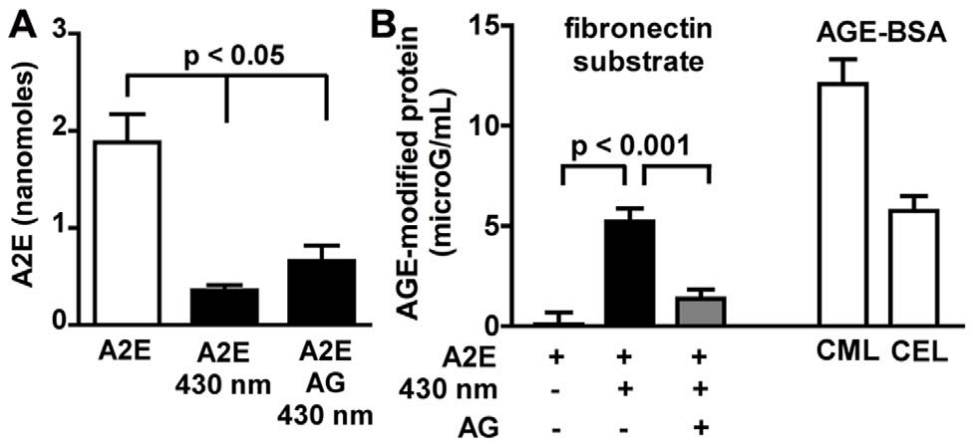
AGE-modification of a fibronectin substrate accompanies photooxidation/photodegradation of intracellular A2E. (*A*) A2E content of ARPE19 is diminished by irradiation (430 nm) in the presence/absence of aminoguanidine (AG). UPLC quantitation; Mean ± SEM of 3 experiments. (*B*) AGE-modification of fibronectin substrate underlying irradiated A2E-containing ARPE19 cells. Prior accumulation of aminoguanidine within the cells reduced AGE-formation. ELISA quantitation as AGE-BSA equivalent units. The anti-AGE antibody recognized both carboxymethyllysine (CML)-BSA and carboxyethyllysine (CEL)-BSA. + presence of condition. Mean ± SEM of 7 experiments.

**Figure 5:**
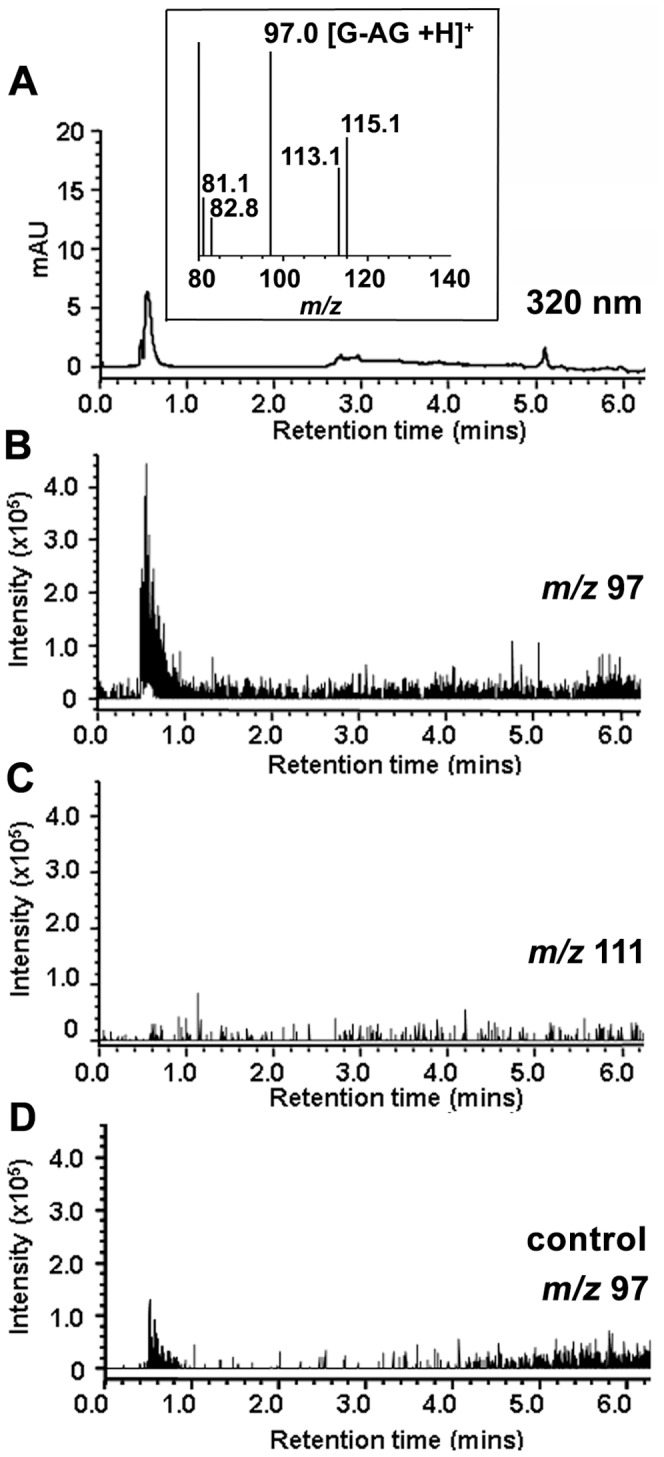
UPLC-MS detection of aminoguanidine (AG)-adducts in extracts of 430 nm irradiated A2E-ARPE19 cells. (*A*) Chromatogram with UV detection at 320 nm. Irradiated A2E-ARPE19 cells. *Inset,* mass spectrum of peak eluting at retention time 0.6 mins. (*B,C*) Selected ion monitoring chromatograms at *m/z* 97 (GO-AG adduct) and *m/z* 111 (MG-AG adduct). Irradiated A2E-ARPE19 cells. (*D*) Selected ion monitoring chromatogram at *m/z* 97. Control nonirradiated A2E-ARPE19 cells.

We next sought to determine whether GO or MG released from photodegraded intracellular A2E could lead to extracellular AGE formation, in this case AGE-modification of a fibronectin substrate on which cultured cells were grown [25]. To that end, we used a culture model wherein the bisretinoid A2E is allowed to accumulate in ARPE-19 cells. We have previously shown that in this model, A2E accumulates in the lysosomal compartment of the cells just as *in vivo* [26]. ARPE-19 cells are preferable for these experiments since in primary cultures of RPE, bisretinoid levels vary. The A2E-containing cells were irradiated at 430 nm and fibronectin was immunoprecipitated from the recovered cells and substrate. The protein samples were adsorbed onto 96-well ELISA plates and the AGE-adducts were probed with an anti-AGE antibody. Conditions included A2E-ARPE19 cells pre-treated with aminoguanidine to allow for intracellular accumulation [27]. As shown in Figure 4, irradiation of A2E-ARPE19 cells resulted in consumption of A2E reflecting A2E photooxidation and photodegradation [22], [28]. The magnitude of the decrease was similar with and without aminoguanidine pre-treatment (Figure 4A). Irradiation of A2E-ARPE19 overlying the fibronectin substrate resulted in substantial AGE-deposition within the extracellular fibronectin when compared to A2E-containing cells that were not irradiated (Figure 4B). Note that the anti-AGE antibody used in the ELISA reacted with both CML-bovine serum albumin (BSA) and CEL-BSA (Figure 4B), indicating an ability of the antibody to recognize forms of AGE produced by both GO and MG, respectively. In the case of A2E-ARPE19 cells that had accumulated aminoguanidine, AGE-fibronectin adduct formation was reduced. The perturbation of AGE-modification could be explained by the ability of intracellular aminoguanidine to scavenge GO and MG as it was generated during photodegradation of A2E within the cells, thus reducing its release into the extracellular milieu. To test for this possibility, we analyzed the cell homogenates for GO- and MG-aminoguanidine adducts. Accordingly, UPLC chromatographic separation (Figure 5A) with MS detection demonstrated a pronounced species at *m/z* 97 indicative of the GO-aminoguanidine adduct (Figure 5B). However we did not detect a compound at *mz* 111 as would be expected for an MG-aminoguanidine conjugate (Figure 5C). Triosephosphates (e.g. glyceraldehyde-3-phosphate) are known to be a cellular source of MG, particularly under conditions of hyperglycemia wherein triosephosphates accumulate [23]. However, in these experiments we negated the latter source as an explanation for the dicarbonyl release by comparison to control nonirradiated A2E-ARPE19 cells.

### Trapping of GO and MG Released into the Extracellular Milieu Following Photodegradation of Intracellular A2E

**Figure 6:**
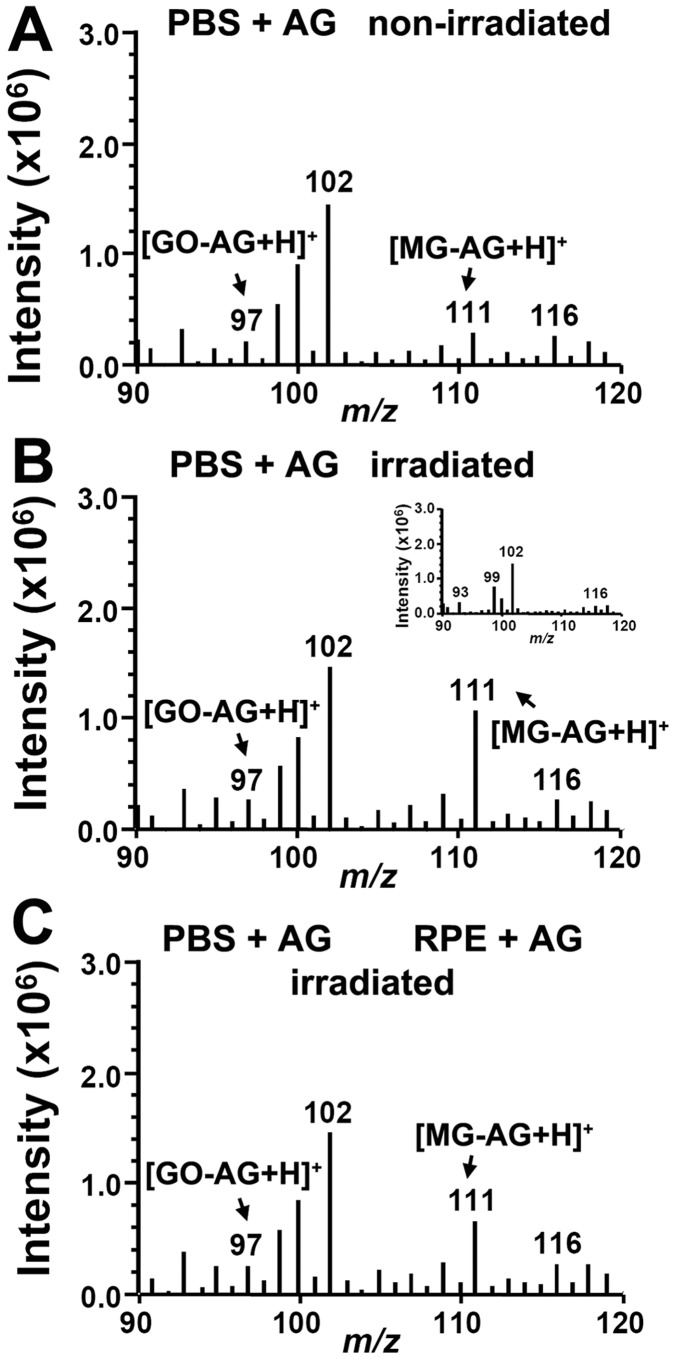
Positive ESI-MS spectra indicating release of methylglyoxal (MG) into the extracellular milieu following photodegradation of intracellular A2E. Pretreatment of the cells with aminoguanidine (RPE-AG) reduces this release. Extracellular trapping by aminoguanidine in PBS (PBS-AG). (*A*) PBS-AG recovered from ARPE19 cells that had accumulated A2E (A2E-ARPE19) and were not irradiated. (*B*) Recovered PBS-AG that had overlaid A2E-ARPE19 cells during irradiation. Note increase in MG-AG adduct (*m/z* 111; [M+H]^+^). *Inset*, ESI-spectra (direct injection) obtained with ethylacetate only. The prominent *m/z* 102 peak attributable to ethylacetate serves as an internal control (*C*) Aminoguanidine-containing PBS that had overlaid irradiated A2E-ARPE19 cells pre-treated with aminoguanidine. GO-AG adduct, *m/z* 97; [M+H]^+^; MG-AG adduct, (*m/z* 111; [M+H]^+^.

AGE-modification of fibronectin under conditions in which intracellular A2E photodegrades, suggests that AGE-eliciting photoproducts such as MG and GO, are liberated from the cells. Thus we next designed experiments to trap MG and GO by aminoguanidine if the dicarbonyls were released from cells upon irradiation of A2E-ARPE19 cells. By positive mode ESI, MG- and GO-aminoguanidine adducts were expected to yield peaks at *m/z* 111 and *m/z* 97 ([M+H]^+^), respectively. As shown in Figure 6, aminoguanidine-containing PBS that had been incubated with non-irradiated A2E-ARPE19 cells exhibited MS signals indicative of background levels of MG-aminoguanidine and GO-aminoguanidine adducts (Figure 6A). The signal for the MG-aminoguanidine adduct (*m/z* 111) was increased within PBS-aminoguanidine that had been incubated with A2E-ARPE19 cells during irradiation (Figure 6B). Intracellular accumulation of aminoguanidine 48 hours prior to irradiation, reduced the external MG-aminoguanidine signal (Figure 6C). We interpret the latter decrease as intracellular scavenging of MG by aminoguanidine as it was generated during photodegradation of A2E within the cells. Aminoguanidine-mediated scavenging would reduce MG release into the extracellular milieu. Note that the *m/z* 102 signal present in all of the MS spectra originates from ethylacetate (Figure 6B, inset) and does not vary in intensity. All samples were reconstituted in ethylacetate before injection into the MS detector; thus this peak served as an internal standard controlling for run-to-run variability in sample injection or instrument response. The GO-aminoguanidine adduct peak (*m/z* 97) was of low intensity and exhibited little change, perhaps due to insufficient detection sensitivity. It is of interest however, that in these experiments, the adduct we detected intracellularly was GO-aminoguanidine (Figure 5), while MG-aminoguanidine was measurable extracellularly. Whether or not these findings reflect differences in the properties of GO and MG (e.g. membrane permeability) remains to be determined.

## Discussion

The bisretinoid compounds that accumulate as autofluorescent lipofuscin in RPE cells originate in photoreceptors cells from inadvertent reactions of all-*trans*-retinal, the latter being generated when photons are absorbed by visual pigment [1]. These photoactive compounds are transferred secondarily to the RPE. All of these compounds are bestowed with two side-arms, each of which bears systems of alternating single and double carbon-carbon bonds. As a consequence, the pigments in this group absorb in both the UV and visible range of the spectrum. These diretinal pigments include but are not limited to the pyridinium bisretinoid A2E, all-*trans*-retinal dimer and its conjugated family members, A2-DHP-PE and glycerophosphoethanolamine (A2-GPE) [1], [29].

RPE bisretinoid pigments are well known to be both photogenerators and quenchers of reactive forms of oxygen [1]. Specifically, the singlet oxygen that is generated when the bisretinoids are excited with short wavelength light, adds to the bisretinoid, oxidizing at carbon-carbon double bonds. Oxidized forms of A2E and all-*trans*-retinal dimer have been identified *in vivo* [30]–[32]. It is at sites of singlet oxygen addition that photocleavage occurs. We have recently demonstrated that photodegradation of bisretinoid produces a complex mixture of aldehyde-bearing fragments of varying molecular size [22]. In the work we currently discuss, we have shown that both A2E and all-*trans*-retinal dimer undergo photodegradation leading to the release of the oxo-aldehydes GO and MG, the latter being major players in AGE-adduct formation. Unlike reactive forms of molecular oxygen, these small aldehydes are rather long lived and can diffuse from their site of origin [33]. Accordingly, in an *in vitro* assay we have detected the liberation of dicarbonyl from the cells and AGE-adduct formation on extracellular fibronectin. The latter AGE-modification was reduced by aminoguanidine-mediated intracellular scavenging. AGE-modification of the fibronectin substrate indicated that MG and GO can be released from the basal surface of the cells, however apical release cannot be excluded. Taken together, these studies implicate RPE bisretinoids as an important source of MG and GO (Figure 1). To the extent that RPE bisretinoids are specific to the latter cell, this source of MG and GO is likely to be unique to the retina.

For some time, glycolysis has been recognized as the major source of MG and GO and under both physiological and hyperglycemic conditions, MG and GO are endogenously produced within cells. MG and GO can exit cells across the plasma membrane, as evidenced for instance, by cross-linking of extracellular proteins such as collagen IV [34]–[36]. In diabetic retinopathy, AGE-modification of extracellular matrix proteins such as fibronectin and laminin, has been shown to lead to over-expression of the proteins, with the resulting basement membrane thickening promoting the progression to acellular capillaries and vascular leakage that is typical of long-term diabetic complications [37], [38]. As compared to oxidative degradation of glucose, direct AGE formation by MG or GO is more efficient, by several orders of magnitude [39]. Reaction of MG and GO with nucleophilic groups in proteins leads to structurally diverse AGE-modifications. The adducts form primarily on arginine and lysine residues and the major products are nonfluorescent hydroimidazolones; the blue fluorescent argpyrimidine; N^e^-CEL and N^e^-CML that form by reaction of MG and GO, respectively, with lysine residues of proteins; methylglyoxal-lysine dimer (MOLD), a cross-linking adduct between two lysine residues; and methyl glyoxal-derived imidazolium cross**-**link (MODIC), a lysine-arginine cross-linking structure. Other uncharacterized AGE adducts are also known to exist [40]. That MG and GO can partake in covalent cross-linking of extracellular proteins is significant, since the collagen of Bruch’s membrane is increasingly cross-linked with age [5]. This change in the extracellular matrix is thought to explain altered properties of Bruch’s membrane such as reduced hydraulic conductivity and permeability, enhanced rigidity and thickening [41]. Cultured RPE grown on an AGE-modified basement membrane substrate exhibits reduced tight junctions and changes in mRNA expression including mRNA that encodes proteins involved in cell attachment and immune responses [42]. Protein cross-linking by AGE-modification can also confer resistance to proteolysis, including that mediated by matrix metalloproteinases [35]. CEL and CML along with pentosidine have all been shown to increase with age in human Bruch’s membrane [14], [15], [17], [18] and are reported to be prominent in both neovascular and atrophic AMD [16], [43].

Does bisretinoid photooxidation and photodegradation occur in vivo? Some lines of evidence indicate that indeed these processes occur in the eye. For instance, mono- and bis-peroxy-A2E, mono- and bis-furano-A2E, mono- and bis-peroxy-all-transretinal dimer and mono- and bis-furano-all-trans-retinal dimer are detected in extracts from human and mouse eyes [28], [31]. The photolysis of bisretinoid at sites of photooxidation could also explain the observation that photooxidized forms of A2E do not accumulate with age [44]. Nevertheless, this is a question that should be addressed in future studies.

Some currently ongoing clinical trials aim to develop treatments for age-related macular degeneration based on limiting RPE bisretinoid lipofuscin formation [45]. The results reported here indicate that therapies such as these may have benefits that extend beyond effects on RPE bisretinoid accumulation alone and that could include preservation of Bruch’s membrane integrity.

## Materials and Methods

### Cells

Confluent human RPE cells (ARPE19; American Type Culture Collection, Manassas, VA) devoid of bisretinoid lipofuscin [26] were allowed to accumulate synthesized A2E [46] into the lysosomal compartment [26] from a 10 microM concentration in media (A2E-ARPE19) [25]. The cells subsequently remained quiescent for 1 week. For some experiments the dishes were coated with fibronectin (10 microG/cm^2^; Invitrogen, Carlsbad, CA) before cell plating. After incubating for 5 days in A2E-free medium, the cells were treated/not treated with aminoguanidine (100 microM in culture medium; 48 hrs; Cayman, Ann Arbor, MI). Transport of aminoguanidine into cells is evidenced by its ability to interrupt intracellular signaling pathways [27], [47]. Before light exposure, culture medium was replaced with phosphate-buffered saline (PBS; with calcium, magnesium and glucose) that contained/did not contain aminoguanidine (100 microM; PBS-AG). Irradiation at 430±30 nm was delivered to the entire area of a 35 mm dish (1 mW/cm^2^, 20 min). PBS- aminoguanidine samples were recovered, concentrated, re-dissolved in ethylacetate (5 microL) and subjected to ultra performance liquid chromatography/mass spectrometry (UPLC/MS) as described below.

### Reaction with 4-nitrophenylhydrazine (4NPH)

Authentic samples of MG and GO (Sigma-Aldrich, St. Louis MO) derivatized with 4NPH (Sigma-Aldrich) were generated as described [22]. After dilution in methanol the sample was subjected to electrospray ionization-mass spectrometry (ESI-MS) analysis. In addition, A2E or all-*trans*-retinal dimer (200 microM in 200 microL water with 1% DMSO) were irradiated (430±20 nm, 1.3 mW/cm^2^; 30 min for A2E and 15 min for all-*trans*-retinal dimer). Samples were dried under argon and pooled, dissolved in 200 microL ethanol, and then mixed with 200 microL of 200 µM 4NPH with 400 microL of glacial acetic acid and stirred for 2 hours at 60°C. Aliquots (10 microL) of the latter mixture were added to acetonitrile (100 microL) and 5 microL samples were analyzed by negative ESI-MS using a Waters Acquity Quadrupole (SQD) mass spectrometer (MS). The capillary voltage was set to 3.0 KV and the cone voltage was set to −30 V.

### Reaction with Aminoguanidine

Authentic MG- and GO-aminoguanidine adducts were generated by incubating aminoguanidine (6 mM; Sigma-Aldrich) with MG and GO (0.14 mmol in PBS) at 37°C for 10 min. A2E or all-*trans*-retinal dimer (200 microM in DBPS with 1% DMSO) were irradiated (430±20 nm, 1.3 mW/cm^2^; 30 min for A2E and 15 min for all-*trans*-retinal dimer) and then incubated (1 hour, 37°C, with stirring) with aminoguanidine (3 mM). Alternatively, A2E or all-*trans*-retinal dimer were first combined with aminoguanidine then irradiated and the mixture was stirred for 30 minutes (37°C). All samples were concentrated and re-dissolved in ethylacetate and prepared in 50% acetonitrile/methanol (1∶1 v/v, with 0.1% formic acid) with 50% water (with 0.1% formic acid) for direct injection positive ESI-MS (capillary voltage, 3.0 KV; cone voltage, 30 V). Authentic 3-amino 1,2,4-triazine (1 mM in ethylacetate; Sigma-Aldrich), the product of reaction of AG and GO [24], was also analyzed by direct injection ESI-MS.

### Detection of A2E and Aminoguanidine-adducts in RPE

A Waters Acquity ultra performance liquid chromatography (UPLC) system (Waters, New Jersey, USA) was operated with a Waters SQD single quadrupole mass spectrometer (electrospray ionization mode, ESI). PDA detection at 320 nm; and an Xbridge® C18 column (2.5 µm, 3.0×50 mm I.D.) were used. Chromatographic separation was performed using a gradient of acetonitrile/methanol (1∶1) in water with 0.1% formic acid and flow rate of 0.5 mL/min. For aminoguanidine-adduct detection, a concentrated extract in 100% methanol was delivered as a 5 microL injectant and the gradient used was 0% (0–1 min) acetonitrile/methanol; 0–98% (1–10 min) acetonitrile/methanol; 98% (10–12 min) acetonitrile/methanol. To quantify A2E, the gradient was 70–85% acetonitrile/methanol (0–60 min).

### ELISA

The cells and substrate were harvested by scraping and fibronectin was immunoprecipitated from the lysate using rabbit polyclonal antibody to fibronection (ABCAM Inc, Cambridge, MA) and protein A-Agarose (Roche Diagnostics GmbH, Germany). Protein concentrations were measured with Bio-Rad protein assay kit (Bio-Rad Laboratories, Hercules, CA) and samples were adjusted to 10 microG/mL total protein with PBS. Advanced glycation end product (AGE) was measured using the AGE ELISA kit (Cell Biolabs, Inc. San Diego, CA). Unknown samples (10 microG/mL), CML-modified bovine serum albumin (BSA) (10 microG/mL; CycLex Ltd, Nagana Japan), CEL-modified BSA (10 microG/mL; CycLex Ltd) and AGE-BSA standards (Cell Biolabs) were loaded into 96-well protein binding plate in duplicate and incubated at 4°C overnight. After incubating in the diluent buffer followed by anti-AGE antibody and HRP-conjugated second antibody, absorbance was read at 450 nm and background values (BSA or stock fibronectin, as appropriate) were subtracted to control for extraneous sources of AGE in the analyte. Absorbance readings were converted to microG/ml by comparison to a standard curve constructed from known amounts of AGE-BSA.

## Supporting Information

Figure S1 **Glyoxal (GO) released by photodegradation of A2E exhibits the same m/z signal (** ***m/z*** **97) as authentic 3-amino-1,2,4 triazine.** (*A*) Positive ESI-MS spectrum in the range of *m/z* 90–120 to detect commercially obtained 3-amino-1,2,4 triazine (1 mM). (*B*) GO-AG adduct (*m/z* 97; [M+H]^+^) generated by reaction of authentic GO (3 mM) with aminoguanidine (AG) (6 mM; AG-bicarbonate). (*C*) Irradiation of a mixture of A2E (200 microM) and AG (6 mM; AG-bicarbonate) generates GO-AG adduct (*m/z* 97) and MG-AG adduct (*m/z* 111).
(TIF)
