# Supplementary figures and images for: A Novel Source of Methylglyoxal and Glyoxal in Retina: Implications for Age-Related Macular Degeneration
Source: PLoS One. 2012 Jul 19;7(7):e41309. doi: 10.1371/journal.pone.0041309 (PMC3400616; doi:10.1371/journal.pone.0041309)

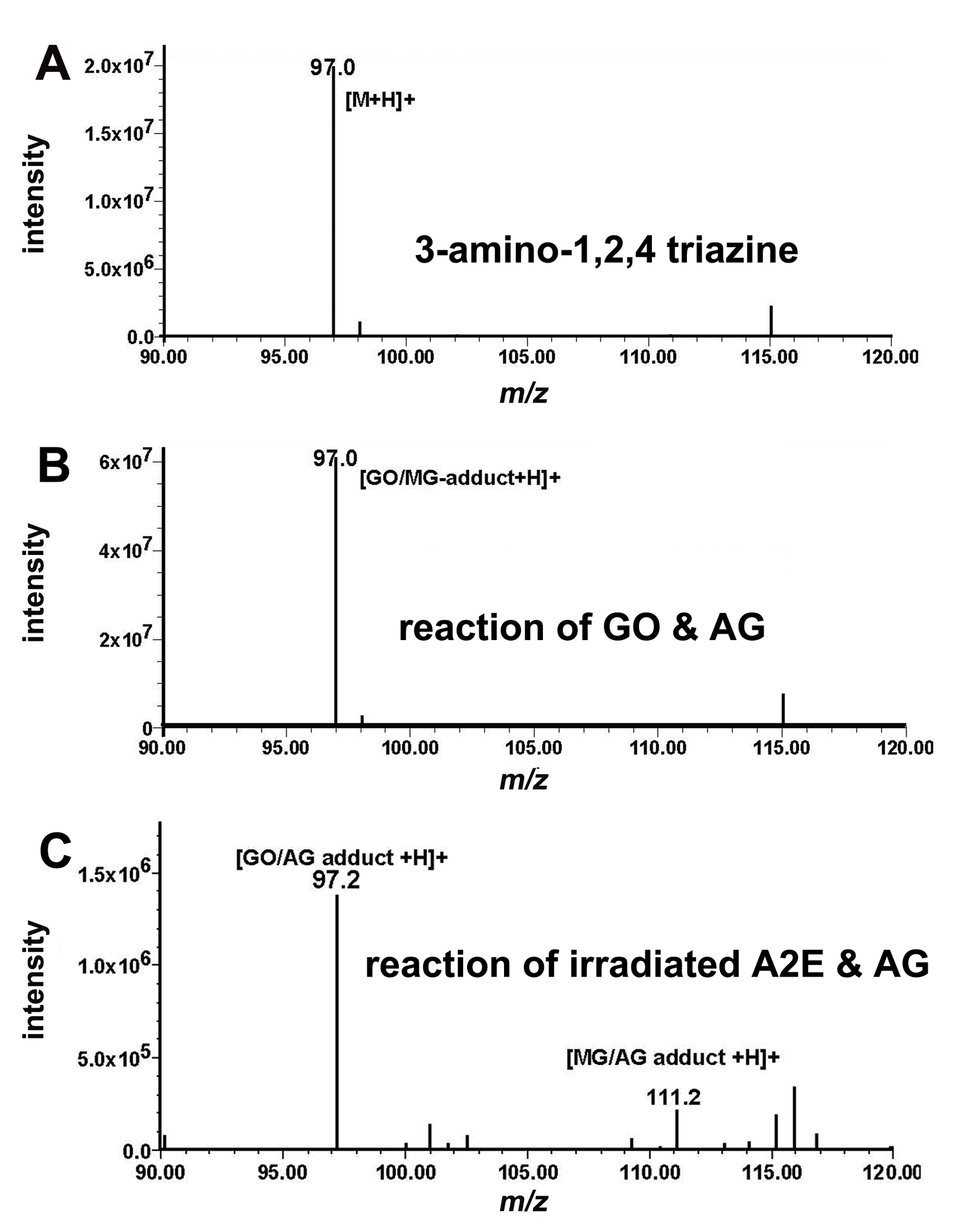

Supplement: Figure S1 — Glyoxal (GO) released by photodegradation of A2E exhibits the same m/z signal ( m/z 97) as authentic 3-amino-1,2,4 triazine. (A) Positive ESI-MS spectrum in the range of m/z 90–120 to detect commercially obtained 3-amino-1,2,4 triazine (1 mM). (B) GO-AG adduct (m/z 97; [M+H]+) generated by reaction of authentic GO (3 mM) with aminoguanidine (AG) (6 mM; AG-bicarbonate). (C) Irradiation of a mixture of A2E (200 microM) and AG (6 mM; AG-bicarbonate) generates GO-AG adduct (m/z 97) and MG-AG adduct (m/z 111). (TIF) [file pone.0041309.s001.tif]
